# ASPM Is a Prognostic Biomarker and Correlates With Immune Infiltration in Kidney Renal Clear Cell Carcinoma and Liver Hepatocellular Carcinoma

**DOI:** 10.3389/fonc.2022.632042

**Published:** 2022-04-20

**Authors:** Tingting Deng, Yang Liu, Jialang Zhuang, Yizhe Tang, Qin Huo

**Affiliations:** Department of Otolaryngology and Geriatric Medicine, Guangdong Key Laboratory of Systems Biology and Synthetic Biology for Urogenital Tumors, Shenzhen Second People’s Hospital, First Affiliated Hospital of Shenzhen University, Health Science Center, Shenzhen University, Shenzhen, China

**Keywords:** ASPM, gene expression, prognosis, tumor-infiltrating, biomarker

## Abstract

**Background:**

Abnormal spindle microtubule assembly (ASPM) is a centrosomal protein and that is related to a poor clinical prognosis and recurrence. However, the relationship between ASPM expression, tumor immunity, and the prognosis of different cancers remains unclear.

**Methods:**

ASPM expression and its influence on tumor prognosis were analyzed using the Tumor Immune Estimation Resource (TIMER), UALCAN, OncoLnc, and Gene Expression Profiling Interactive Analysis (GEPIA) databases. The relationship between ASPM expression and tumor immunity was analyzed using the TIMER and GEPIA databases, and the results were further verified using qPCR, western blot, and multiplex quantitative immuno fluorescence.

**Results:**

The results showed that ASPM expression was significantly higher in most cancer tissues than in corresponding normal tissues, including kidney renal clear cell carcinoma (KIRC), kidney renal papillary cell carcinoma (KIRP), liver hepatocellular carcinoma (LIHC), lung adenocarcinoma (LUAD), pancreatic adenocarcinoma (PAAD), and breast invasive carcinoma (BRCA). ASPM expression was significantly higher in late-stage cancers than in early-stages cancers (e.g., KIRC, KIRP, LIHC, LUAD, and BRCA; p < 0.05), demonstrating a possible role of ASPM in cancer progression and invasion. Moreover, our data showed that high ASPM expression was associated with poor overall survival, and disease-specific survival in KIRC and LIHC (p < 0.05). Besides, Cox hazard regression analysis results showed that ASPM may be an independent prognostic factor for KIRC and LIHC. ASPM expression showed a strong correlation with tumor-infiltrating B cells, CD8^+^ T cells, and M2 macrophages in KIRC and LIHC.

**Conclusions:**

These findings demonstrate that the high expression of ASPM indicates poor prognosis as well as increased levels of immune cell infiltration in KIRC and LIHC. ASPM expression may serve as a novel prognostic biomarker for both the clinical outcome and immune cell infiltration in KIRC and LIHC.

## Introduction

Tumor-infiltrating lymphocytes are mononuclear immune cells that infiltrate tumor tissue (1). Immune cells are composed of multiple lymphocytes, such as CD4^+^ T cells, CD8^+^ T cells, B cells, macrophages, natural killer (NK) cells, and dendritic cells (DCs) (2). Studies have shown that tumor-infiltrating immune cells are closely related to clinical outcomes (3), including those of ovarian cancer (4), triple-negative breast cancer (5), colorectal cancer (6), esophageal cancer (7), and hepatocellular carcinoma (8). Uryvaev A et al. demonstrated that immune cells can be used as predictive biomarkers for the anti-PD1 treatment response in patients with metastatic non-small cell lung cancer or metastatic melanoma (9). Kashiwagi S et al. revealed that the application of immune cells to monitor the antitumor immune response may be a useful indicator for predicting the efficacy of TPD chemotherapy for HER2-positive breast cancer (10). In addition, studies have shown that tumor-infiltrating CD8^+^ T cells expressing PD-1 can improve the efficacy of adoptive T cell therapy (11). However, the clinical impact of immune cells on many cancers remains poorly understood. Therefore, it is essential to explore the molecular mechanisms involved in pan-cancer pathogenesis to identify novel prognostic biomarkers and potential therapeutic targets for cancers.

Abnormal spindle microtubule assembly (ASPM) is ubiquitously expressed in the ventricular region of the posterior cranial fossa and plays a vital role in the regulation of the mitotic spindle, neurogenesis, and brain size (12–14). In recent years, an increasing number of studies have shown that ASPM is highly expressed not only in central nervous system tumors, but also in a variety of solid tumors, such as prostate cancer (15), breast cancer (16), and bladder cancer (17). Pai et al. (18) reported that ASPM promotes the metastasis and progression of prostate cancer by enhancing the Wnt-Dvl-3-β-catenin signaling pathway. Recent evidence has shown that ASPM promotes the growth of glioblastoma by regulating the progression of G1 restriction points and Wnt-β-catenin signaling (19). Kidney renal clear cell carcinoma (KIRC) and kidney renal papillary cell carcinoma (KIRP) are a significant cause of cancer-related deaths (20, 21). At present, no study has found a reliable treatment to improve the survival of patients. Liver hepatocellular carcinoma (LIHC) is the most prevalent primary cancer of the liver. So far, there is still no precise biomarker that predicts response to immunotherapy in LIHC (22). A study reported that ASPM has previously been reported as a potential prognostic biomarker for bladder cancer. It may also be a potential immunotherapeutic target with future clinical significance (23). However, the relationship between ASPM and clinical prognosis and immune cell infiltration in other cancers patients remains unclear.

In the present study, we investigated the expression pattern of ASPM by using the Tumor Immune Estimation Resource (TIMER) and UALCAN databases. The functional protein association network and functional enrichment were analyzed using the STRING and Enrichr databases. The correlation between the expression of ASPM and patient survival was calculated by the OncoLnc and Gene Expression Profiling Interactive Analysis (GEPIA) databases. To determine any potential correlation of ASPM expression with immune cell infiltration in cancers, Spearman’s correlation was used to evaluate samples from the TIMER and GEPIA databases. The results of this report reveal the important role of ASPM in cancer and provide an underlying mechanism and potential relationship between ASPM and tumor immunity.

## Methods

### Patient Samples

This study was performed on archived tissues from 20 diagnosed cases of LIHC and 20 control samples (muscle, lymph nodes), as well as 20 diagnosed cases of LIHC and 20 control samples (muscle, lymph nodes).These samples were obtained from Shenzhen Second People’s Hospital. This study was approved by the Ethics Committee of Shenzhen Second People’s Hospital in accordance with the principles of the Declaration of Helsinki.

### ASPM Gene Expression Analysis

The expression levels of *ASPM* in several cancers were determined from the TIMER database (https://cistrome.shinyapps.io/timer/), It includes 10,897 samples for 32 cancer types from The Cancer Genome Atlas (TCGA) database to estimate the role of immune infiltration (24). TIMER uses RNA-Seq expression profiling data to detect immune cell infiltration in tumor tissue based on statistical analysis of gene expression profiles (25). Besides, we further determined *ASPM* expression using the UALCAN database (http://ualcan.path.uab.edu/), which is an effective online analysis and mining website for an in-depth analysis of gene expression data using TCGA levels 3 RNA-seq and clinical data from 31 cancer types (26). In this study we analyzed the differential expression of *ASPM* from various cancers and investigated *ASPM* expression on the basis of tumor grade.

### Construction of the PPI Network and Functional Enrichment Analysis

We analyzed the functional protein association network by using the STRING database (https://string-db.org/) (27). Protein name: ASPM and organism: *Homo sapiens* were analyzed. The nodes and edges in the network represent the target genes and their interactions, respectively (28). Subsequently, Gene Ontology (GO) analysis (BP: biological process, CC: cellular component, and MF: molecular function) was performed using Enrichr (http://amp.pharm.mssm.edu/Enrichr), which is a comprehensive tool for gene enrichment analysis (29).

### Survival Analysis

The correlation between *ASPM* and survival of patients with KIRC, KIRP, LIHC, LUAD, PAAD, and BRCA was assessed by OncoLnc (http://www.oncolnc.org/), which provides an online database of TCGA survival data related to mRNA, miRNA, and lncRNA expression levels and was used to investigate their prognostic values (30, 31). For the accurate interpretation of *ASPM* expression detection results, we set the cut-off value as 50%. We also analyzed the prognostic value of *ASPM* expression in KIRC, KIRP, LIHC, LUAD, PAAD, and BRCA tissue using the GEPIA database (http://gepia.cancer-pku.cn/index.html), an online database used to evaluate the correlation between *ASPM* expression and clinicopathologic information in cancers (32). We compared data from 1085 breast tumor samples with 291 and normal group samples. We used the “Survival” module of GEPIA to evaluate the correlation of ASPM expression with the prognosis of KIRC, KIRP, LIHC, LUAD, PAAD, and BRCA.

### Correlation Analysis of *ASPM* and Immune Cell Infiltration

The abundance of tumor-infiltrating immune cells in KIRC, KIRP, LIHC, LUAD, and PAAD tissues was predicted using the TIMER database. We determined the level of *ASPM* gene expression and the abundance of infiltrating immune cells (B cells, CD4^+^ T cells, CD8^+^ T cells, neutrophils, macrophages, and dendritic cells). We explored the expression of ASPM and the genetic markers of immune cell subsets through the correlation module in the TIMER database, calculated Spearman’s correlation coefficient, and estimated the statistical significance (33).

### Immunohistochemistry

We used archived tissues from 20 diagnosed cases of LIHC and 20 control samples (muscle, lymph nodes), as well as 20 diagnosed cases of LIHC and 20 control samples (muscle, lymph nodes) for immunohistochemistry. To validate the relationship between ASPM expression and B cells (marker: CD19), CD8^+^ T cells (marker: CD8A), and M2 macrophages (marker: CD163), we performed immunohistochemistry to assess ASPM, CD19, CD8A, and CD163. The concentration of the four antibodies was optimized; the following primary antibodies were used: rabbit anti-ASPM (1:100, Affinity Biosciences), anti-CD19 (1:100, Affinity Biosciences), anti-CD8A (1:100, Affinity Biosciences), and anti-CD163 (1:100, Affinity Biosciences). All of the above fresh tissue was under cryopreservation and processed into frozen sections. The slides were deparaffinized and rehydrated through graded alcohols and were then subjected to an antigen retrieval procedure (10 mM sodium citrate, 0.05% Tween-20, pH 6.0 for 25 min). After treatment with 3% H_2_O_2_ for 10 min to quench endogenous peroxidase activity. Then the tissues were incubated by primary antibodies and incubated at 4 ˚C overnight and incubated by secondary antibodies. We defined an appropriate scoring system based on the immune response scoring method proposed by Remmele and Stegner, using the product of staining intensity and the percentage of positive cells. The staining intensity is divided into 4 levels, that is, level 0 is negative if no positive cells are seen, level 1 is weakly positive, level 2 is moderately positive, and level 3 is strong positive. The percentage of positive cells is divided into 5 levels, that is, level 0 is negative, level 1 ≤ 10%, level 2 is 11%-50%, level 3 is 51%-80%, and level 4 > 80%. The expression density of ASPM, CD8A, CD19, and CD163 was quantitated by scoring staining intensity through automated analysis, including negative (–), weak (+), moderate (++), and strong (+++) staining, respectively. IHC scores were graded by manual interpretation by three statisticians and three pathology experts.

### Multiplex Quantitative Immunofluorescence

We reused previously collected 20 diagnosed cases of KIRC and 20 diagnosed cases of LIHC samples for ultiplex quantitative immunofluorescence staining was obtained using pano 7-plex IHC kit, cat 000410100 (panovue, Beijing, China). We performed multiplex quantitative immunofluorescence to assess the relationship between ASPM expression and B cells, CD8^+^ T cells, and M2 macrophages. The following primary antibodies were used: rabbit anti-ASPM (1:100, Affinity Biosciences), anti-CD19 (1:100, Affinity Biosciences), anti-CD8A (1:100, Affinity Biosciences), and anti-CD163 (1:100, Affinity Biosciences). Different primary antibodies were applied in turn, and then incubated with horseradish peroxidase bound secondary antibodies and tyramine signal amplification (TSA) technology for high-resolution subcellular localization of proteins. After TSA, the slides were subjected to microwave heat treatment. All human antigens were labeled and stained with 4’-6’-diamino-2-phenylindole (DAPI, Sigma-Aldrich). Stained slides were scanned using a mantra system (PerkinElmer, Waltham, Massachusetts, US), which captured fluorescence spectra at 20 nm wavelength intervals at the same exposure time; the scans are combined to form a single laminated image.

### Western-Blot Analysis and Quantitative Real-Time PCR

In this study, we reused previously collected 10 diagnosed cases of KIRC and 10 diagnosed cases of LIHC archived tissues for western blot and RT-qPCR analysis, these tissues which do have ethics. This study was approved by the Ethics Committee of Shenzhen Second People’s Hospital in accordance with the principles of the Declaration of Helsinki. We extracted proteins with phosphate-safe extraction reagent.

The protein was separated using 10% SDS-PAGE and was then transferred to a polyvinylidene fluoride (PVDF) membrane and blocked using 5% skimmed milk in triethanolamine-buffered saline solution (TBS) at 25°C for 1 h, incubated with specific primary ASPM antibodie (DF10064, 1:200 dilution, Affinity) overnight at 4°C and then incubated with secondary antibodies for 2 h at 37°C. Images of protein bands were captured by ECL substrate and ImageQuant RT ECL System (GE Healthcare). The images were analyzed using Quantity One software.

We performed RNA extraction from the above-collected tissue (which has been ethically approved). The total RNA was extracted by using the RNeasy Mini Kit (QIAGEN). Total RNA was then reverse-transcribed using Script II RT Kit (QIAGEN). Samples were quantified following the instructions of SYBR^®^ Green PCR Kit (QIAGEN). The primers for detection of ASPM (F:GAGACCTTGGTGGAATACCTGC,R:ACGAAGATCCAAAAGCCTTGCAC) and GAPDH (F: CTGGAACGGTGAAGGTGAC,R:AAGGGACTTCCTGTAACAATGCA) were synthesized by Sangon Biotech (Shanghai, China).

### Statistical Analysis

Survival curves were generated through analysis on OncoLnc online tool and GEPIA database. The data were processed using a one-way analysis of variance (ANOVA). Data were presented as the means of the results from at least three independent experiments. Overall survival (OS) was calculated using Kaplan-Meier analysis and log-rank test. Use univariate Cox analysis to screen potential prognostic factors, and multivariate Cox analysis to verify the effect of ASPM expression on survival along with other clinical variables. A value of p < 0.05 was considered statistically significant.

## Results

### *ASPM* mRNA Expression Levels in Various Types of Cancer

**Figure 1:**
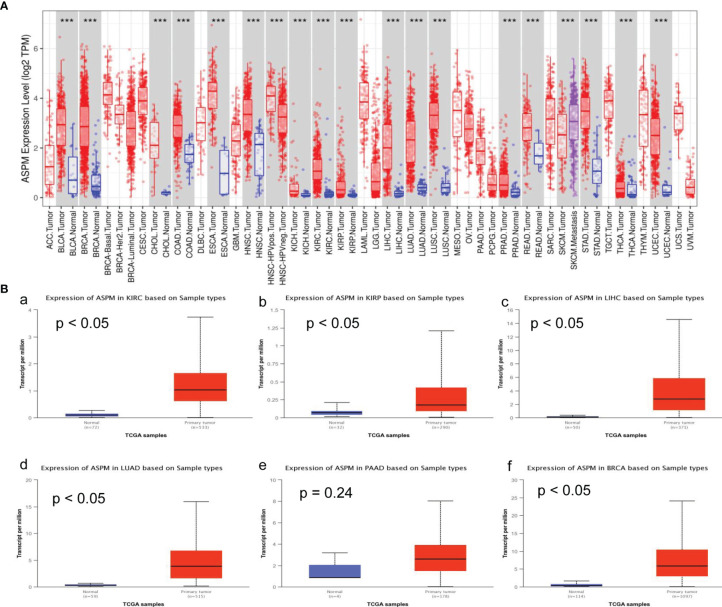
ASPM mRNA expression levels in various types of cancer. **(A)** The expression levels of ASPM in various types of cancer tissues and their corresponding normal tissues were analyzed using the Tumor Immune Estimation Resource (TIMER) database. It includes 10,897 samples for 32 cancer types from The Cancer Genome Atlas (TCGA) database to estimate the role of immune infiltration. The color intensity (red or blue) is directly proportional to the significance level of upregulation or downregulation, respectively. The expression of ASPM was upregulated in BLCA (bladder urothelial carcinoma), BRCA (breast invasive carcinoma), CHOL (cholangiocarcinoma), COAD (colon adenocarcinoma), ESCA (esophageal carcinoma), HNSC (head and neck squamous cell carcinoma), KICH (kidney chromophobe), KIRC (kidney renal clear cell carcinoma) and KIRP (kidney renal papillary cell carcinoma), LIHC (Liver hepatocellular carcinoma), LUAD (lung adenocarcinoma), LUSC (lung squamous cell carcinoma), PRAD (prostate adenocarcinoma), READ (rectum adenocarcinoma), SKCM (skin cutaneous melanoma), STAD (stomach adenocarcinoma), THCA (thyroid carcinoma), and UCEC (uterine corpus endometrial carcinoma). P-value Significant Codes: 0 ≤ ***< 0.001 ≤ **< 0.01 ≤ *< 0.05 ≤. < 0.1. **(B)** The expression levels of ASPM in different tumor types using the UALCAN database. KIRC: primary tumor (n = 533), normal (n = 72); KIRP: primary tumor (n = 290), normal (n = 32); LIHC: primary tumor (n = 371), normal (n = 50); LUAD: primary tumor (n = 515), normal (n = 59); PAAD: primary tumor (n = 178), normal (n = 4); BRCA: primary tumor (n = 1097), normal (n = 114). **a:** The expression of ASPM in KIRC; **b:** The expression of ASPM in KIRP; **c:** The expression of ASPM in LIHC; **d:** The expression of ASPM in LUAD; **e:** The expression of ASPM in PAAD; **f:** The expression of ASPM in BRCA. n: Number of samples.

To obtain insight into the potential role of *ASPM* in cancer progression, the mRNA levels of *ASPM* in various types of cancer tissues and their corresponding normal tissues were analyzed using the TIMER database. As shown in **Figure 1A**, compared with normal tissues, the expression of *ASPM* was upregulated in BLCA (bladder urothelial carcinoma), BRCA (breast invasive carcinoma), CHOL (cholangiocarcinoma), COAD (colon adenocarcinoma), ESCA (esophageal carcinoma), HNSC (head and neck squamous cell carcinoma), KICH (kidney chromophobe), KIRC, KIRP, LIHC, LUAD (lung adenocarcinoma), LUSC (lung squamous cell carcinoma), PRAD (prostate adenocarcinoma), READ (rectum adenocarcinoma), SKCM (skin cutaneous melanoma), STAD (stomach adenocarcinoma), THCA (thyroid carcinoma), and UCEC (uterine corpus endometrial carcinoma). Additionally, to confirm the differential expression of ASPM between tumor and normal tissues, *ASPM* expression was analyzed using the UALCAN database, and we found that *ASPM* mRNA expression was significantly higher in KIRC (p < 0.05), KIRP (p < 0.05), LIHC (p < 0.05), LUAD (p < 0.05), PAAD (pancreatic adenocarcinoma, p = 0.24), and BRCA (p < 0.05) tissues than in normal tissues (**Figure 1B**).

### Relationship Between *ASPM* Expression and Clinicopathological Parameters

**Figure 2:**
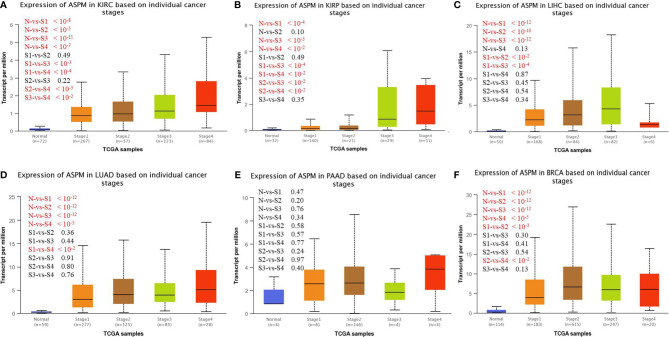
UALCAN analysis for the correlation between ASPM mRNA expression level based on cancer stage. KIRC: Normal (n = 72), stage 1 (n = 267), stage 2 (n = 57), stage 3 (n = 123), stage 4 (n = 84); KIRP: Normal (n = 50), stage 1 (n = 168), stage 2 (n = 84), stage 3 (n = 82), stage 4 (n = 6); LUAD: Normal (n = 59), stage 1 (n = 277), stage 2 (n = 125), stage 3 (n = 85), stage 4 (n = 28); PAAD: Normal (n = 4), stage 1 (n = 6), stage 2 (n = 146), stage 3 (n = 4), stage 4 (n = 4); BRCA: Normal (n = 114), stage 1(n = 183), stage 2(n = 615), stage 3(n = 247), stage 4 (n = 20). **(A)** Expression of ASPM in KIRC based on individual cancer stages. **(B)** Expression of ASPM in KIRP based on individual cancer stages. **(C)** Expression of ASPM in LIHC based on individual cancer stages. **(D)** Expression of ASPM in LUAD based on individual cancer stages. **(E)** Expression of ASPM in PAAD based on individual cancer stages. **(F)** Expression of ASPM in BRCA based on individual cancer stages. n: Number of samples.

We investigated *ASPM* expression on the basis of histological subtype, tumor grade, and other patient conditions using the UALCAN database. The results showed increased expression levels of *ASPM* in all grades of KIRC tissues compared to normal tissues (**Table 1**). Higher *ASPM* expression in clear cell type A (ccA) (p < 10^−8^) and clear cell type B (ccB) (p < 10^−13^) subtype than that in the normal subtype, *ASPM* expression in ccB subtype was significant higher than in ccA subtype (p < 10^−4^). In KIRP tumors, the increase was significant for the CpG island methylator phenotype (CIMP). **ASPM** overexpression was more significant for type 2 papillary renal cell carcinoma (RCC) than for type1 papillary RCC (p < 10^−3^). The expression of *ASPM* was higher in all LIHC tumor tissues than in normal tissues, with that in grade 3 tissues being statistically significant. We found that *ASPM* expression in grade 3 was significant high in grade 1 and grade 2 (p < 10^−4^), but the change in *ASPM* expression between grade 3 and grade 4 for LIHC was not significant. Regarding lung cancers, all histological subtypes of LUAD tumors showed a notable increase in *ASPM* increase, which was more statistically significant for lung adenocarcinoma-not otherwise specified and lung adenocarcinoma mixed subtype (Mixed) than lung bronchioloalveolar carcinoma mucinous (LBC-Mucinous). Regarding patients’ smoking habits, all histological subtypes of LUAD tumor showed increased *ASPM* expression compared with normal tissues, we found that *ASPM* expression was higher in patients who smoked longer (p < 10^-6^). Concerning PAAD tumors, on the basis of tumor grade, *ASPM* expression was higher in all tumor tissues than in normal tissues (**Table 1**), and the increase was statistically significant for PAAD in grade 3. Moreover, the expression of ASPM was higher in all different subtypes, including luminal breast cancer, HER2 positive breast cancer, and triple- negative breast cancer (TNBC). *ASPM* expression in TNBC was higher than that in luminal (p < 10^-13^) and HER2 positive (p < 10^-4^) breast cancer. *ASPM* expression showed the most significant increase in infiltrating ductal carcinoma and infiltrating lobular carcinoma. The expression of *ASPM* was higher in pre-menopause, peri-menopausal, and post-menopause women than in normal women (**Table 1**). We next investigated *ASPM* expression based on cancer stage and found that the expression levels of *ASPM* were significantly higher in late-stage cancers than in early stages of cancer for KIRC, KIRP, LIHC, LUAD and BRCA (**Figure 2**, p-values < 0.05).The expression levels of *ASPM* were significantly higher in stage 3 than stage 1(p < 10^-3^), stage 4 than stage 2 (p < 10^-3^), stage 4 than stage 3 (p < 10^-2^) in KIRC. For KIRP, the expression levels of *ASPM* were significantly higher in stage 3/4 than stage 1/2. The expression levels of *ASPM* were significantly higher in stage 2 than stage 1 for LIHC (p < 10^-2^) and BRCA (p < 10^-3^). However, there is no association in PAAD. These results demonstrate a possible role of *ASPM* in cancer progression and invasion.

**Table 1:** Statistically significant ASPM overexpression based on histological, molecular subtypes, and different patient statuses (only findings with p-value < 0.05 are given).

Tumor	Histological Subtypes	Molecular Subtypes	Tumor Grade	Other Patient Conditions
KIRC		Normal-vs-ccA subtype: p < 10^−8^ ; Normal-vs-ccB subtype: p < 10^−13^; ccA subtype-vs-ccB subtype: p < 10^−4^	Normal-vs-Grade 1:p < 10^−3^ ; Normal-vs-Grade 2: p < 10^−9^; Normal-vs-Grade 3: p < 10^−12^ ; Normal-vs-Grade 4: p < 10^−6^ ; Grade 1-vs-Grade 2: p < 10^−3^ ; Grade 1-vs-Grade 3: p < 10^−5^ ; Grade 1-vs-Grade 4: p < 10^−4^; Grade 2-vs-Grade 3: p < 10^−2^; Grade 2-vs-Grade 4: p < 10^−3^; Grade 3-vs-Grade 4: p < 10^−2^	
KIRP	Normal-VS-Type1 PRCC: p < 10^−5^; Normal-VS-Type2 PRCC: p < 10^−5^ ; Normal-VS-KIRP CIMP: p < 10^−2^ ; Normal-VS-Unclassified PRCC: p < 10^−4^ ; Type1 PRCC-VS-Type2 PRCC: p < 10^−3^ ; Type1 PRCC-VS-KIRP CIMP: p < 10^−2^ ; Type2 PRCC-VS-KIRP CIMP: p < 10^−2^; KIRP CIMP-VS-Unclassified PRCC: p < 10^−2^;			
LIHC			Normal-vs-Grade 1: p < 10^−5^ Normal-vs-Grade 2: p < 10^−12^ Normal-vs-Grade 3: p < 10^−12^ Normal-vs-Grade 4: p < 10^−4^ Grade 1-vs-Grade 3: p < 10^−4^ Grade 2-vs-Grade 3: p < 10^−4^	
LUAD	Normal-vs-NOS: p < 10^−12^ Normal-vs-Mixed: p < 10^−15^ Normal-vs-LBC-NonMucinous: p < 10^−4^ Normal-vs-SolidPatternPredominant: p < 10^−2^ Normal-vs-Acinar: p < 10^−5^ Normal-vs-LBC-Mucinous: p < 10^−2^ Normal-vs-Mucinous carcinoma: p < 10^−3^ Normal-vs-Papillary: p < 10^−3^ NOS-vs-Mixed: p < 10^−4^ NOS-vs-LBC-Mucinous: p < 10^−15^ NOS-vs-Mucinous carcinoma: p < 10^−3^ Mixed-vs-Acinar: p < 10^−2^ Mixed-vs-LBC-Mucinous: p < 10^−8^ ClearCell-vs-LBC-NonMucinous: p < 10^−2^ LBC-NonMucinous-vs-LBC-Mucinous: p < 10^−3^ SolidPatternPredominant-vs-LBC-Mucinous: p < 10^−2^ SolidPatternPredominant-vs-Mucinous carcinoma: p < 10^−2^ Acinar-vs-LBC-Mucinous: p < 10^−4^ Acinar-vs-Mucinous carcinoma: p < 10^−2^ LBC-Mucinous-vs-Mucinous carcinoma: p < 10^−2^ LBC-Mucinous-vs-Papillary: p < 10^−2^			Normal-vs-Non smoker: p < 10^−10^ Normal-vs-Smoker: p < 10^−12^ Normal-vs-Reformed smoker1: p < 10^−12^ Normal-vs-Reformed smoker2: p < 10^−12^ Non smoker-vs-Smoker: p < 10^−4^ Non smoker-vs-Reformed smoker2: p < 10^−3^ Smoker-vs-Reformed smoker1: p < 10^−7^ Reformed smoker1-vs-Reformed smoker2: p < 10^−6^
PAAD			Grade 1-vs-Grade 2: p < 10^−3^ Grade 1-vs-Grade 3: p < 10^−6^ Grade 2-vs-Grade 3: p < 10^−2^	
BRCA	Normal-vs-IDC: p < 10^−12^ Normal-vs-ILC: p < 10^−12^ Normal-vs-Mixed: p < 10^−7^ Normal-vs-Other: p < 10^−9^ Normal-vs-Mucinous: p < 10^−4^ Normal-vs-Medullary: p < 10^−4^ IDC-vs-ILC: p < 10^−12^ IDC-vs-Mixed: p < 10^−2^ IDC-vs-Other: p < 10^−3^ IDC-vs-Mucinous: p < 10^−5^ ILC-vs-Other: p < 10^−2^ ILC-vs-Medullary: p < 10^−7^ Mixed-vs-Medullary: p < 10^−4^ Other-vs-Mucinous: p < 10^−2^ Other-vs-Medullary: p < 10^−4^ Mucinous-vs-Medullary: p < 10^−7^ Metaplastic-vs-Medullary: p < 10^−2^	Normal-vs-Luminal: p < 10^−16^ Normal-vs-HER2 Positive: p < 10^−9^ Normal-vs-TNBC: p < 10^−12^ Luminal-vs-HER2 Positive: p < 10^−2^ Luminal-vs-TNBC: p < 10^−13^ HER2 Positive-vs-TNBC: p < 10^−4^		Normal-vs-Pre-Menopause: p < 10^−12^ Normal-vs-Peri-Menopause: p < 10^−8^ Normal-vs-Post-Menopause: p < 10^−12^ Peri-Menopause-vs-Post-Menopause: p < 10^−2^

### Protein-Protein Interaction and Functional Enrichment Analyses

**Figure 3:**
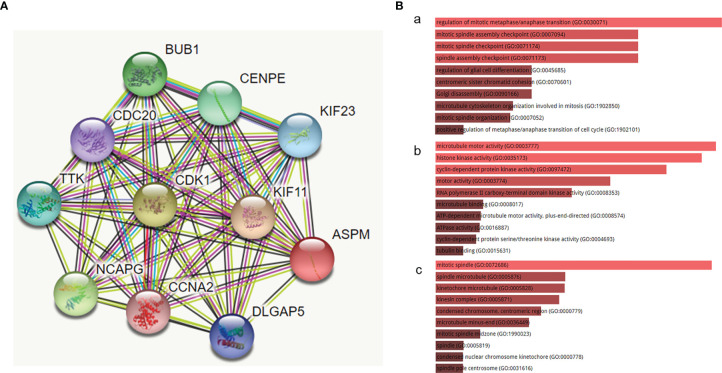
The functional protein association network generated by the STRING database. **(A)** The network of ASPM and its co-expression genes was set up visually. **(B)** The biological functions (GO enrichment) of the identified genes *via* the Enrichr online database. **(a)** biological process, **(b)** molecular function, and **(c)** cellular component. The biological processes of these proteins mainly involved the regulation of mitotic metaphase/anaphase transition (GO:0030071), mitotic spindle assembly checkpoint (GO:0007094), and mitotic spindle checkpoint (GO:0071174). The molecular function term was mainly enriched in microtubule motor activity (GO:0003777), histone kinase activity (GO:0035173), and cyclin-dependent protein kinase activity (GO:0097472). The cell component term was significantly enriched in the mitotic spindle (GO:0072686), spindle microtubule (GO:0005876), and kinetochore microtubule (GO:0005828).

To determine the biological interaction network of ASPM, we analyzed the functional protein association network generated by the STRING database (**Figure 3A**). The coexpression analysis revealed that ASPM was co-expressed with kinesin-like protein KIF11, cyclin-dependent kinase 1 (CDK1), the mitotic checkpoint serine/threonine-protein kinase BUB1, condensin complex subunit 3 (NCAPG), centromere-associated protein E (CENPE), the dual-specificity protein kinase TTK (TTK), the kinesin-like protein KIF23, disks large-associated protein 5 (DLGAP5), cell division cycle protein 20 homolog (CDC20), and Cyclin-A2 (CCNA2), whose correlation scores were 0.995, 0.994, 0.993, 0.993, 0.992, 0.988, 0.987, 0.985, 0.983, and 0.981, respectively.

We examined the biological functions (GO enrichment) of the identified genes *via* the Enrichr online database (**Figure 3B**). GO analysis demonstrated that the biological processes of these proteins mainly involved the regulation of mitotic metaphase/anaphase transition (GO:0030071), mitotic spindle assembly checkpoint (GO:0007094), and mitotic spindle checkpoint (GO:0071174). The molecular function term was mainly enriched in microtubule motor activity (GO:0003777), histone kinase activity (GO:0035173), and cyclin-dependent protein kinase activity (GO:0097472). The cell component term was significantly enriched in the mitotic spindle (GO:0072686), spindle microtubule (GO:0005876), and kinetochore microtubule (GO:0005828). The above biological functions show that the functions of these proteins are closely related to the occurrence and development of cancers.

### Correlation Between *ASPM* Expression and Survival Outcomes

**Figure 4:**
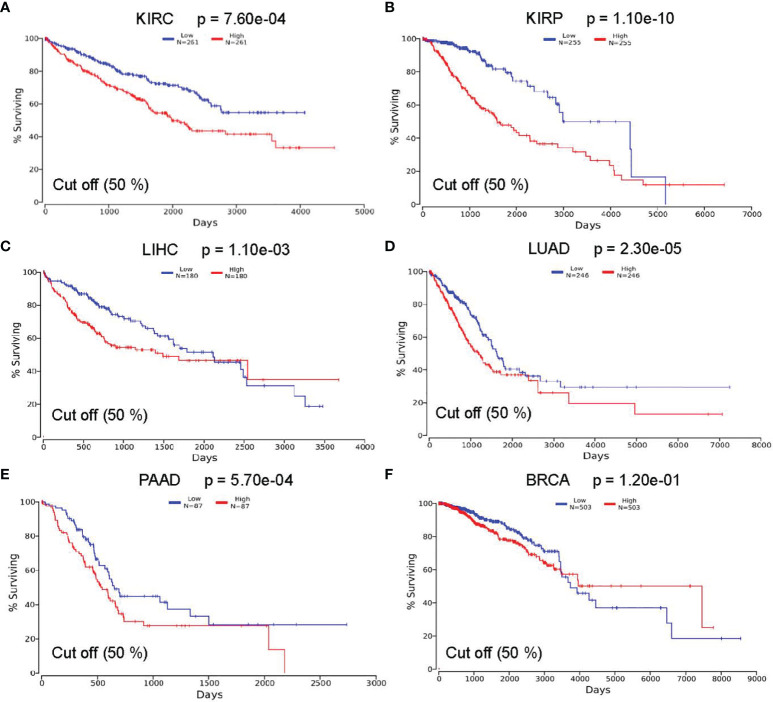
Correlation between ASPM expression and survival outcomes using the OncoLnc online tool. The relationship between the expression level of ASPM and the survival rate of **(A)** KIRC (Low: n = 261; High: n = 261); **(B)** KIRP (Low: n = 255; High: n = 255); **(C)** LIHC (Low: n = 180; High: n = 180); **(D)** LUAD (Low: n = 246; High: n = 246); **(E)** PAAD(Low: n = 87; High: n = 87); and **(F)** BRCA (Low: n = 503; High: n = 503) patients.

To investigate whether the survival rate of KIRC, KIRP, LIHC, LUAD, PAAD, and BRCA patients is related to the expression level of *ASPM*, we compared the overall survival rate of patients with a high expression level of *ASPM* (50%) to those with a low expression level of *ASPM* (50%) using the OncoLnc online tool (**Figure 4**). The results of this database showed the relationship with survival after regression analysis and multivariate analysis performed controlling for other relevant variables. Notably, *ASPM* expression significantly affected the prognosis of different cancers. The relationship between *ASPM* and prognosis was statistically significant, and the overexpression of *ASPM* was related to poor OS. High *ASPM* expression was associated with a poor prognosis in KIRC (p = 7.60e-04), KIRP (p = 1.10e-10), LIHC (p = 1.10e-03), LUAD (p = 2.30e-05), and PAAD (p = 5.70e-04). However, there was no significant correlation between *ASPM* expression and the survival rates in BRCA patients (p = 1.20e-01), which were used as negative controls in subsequent analyses.

**Figure 5:**
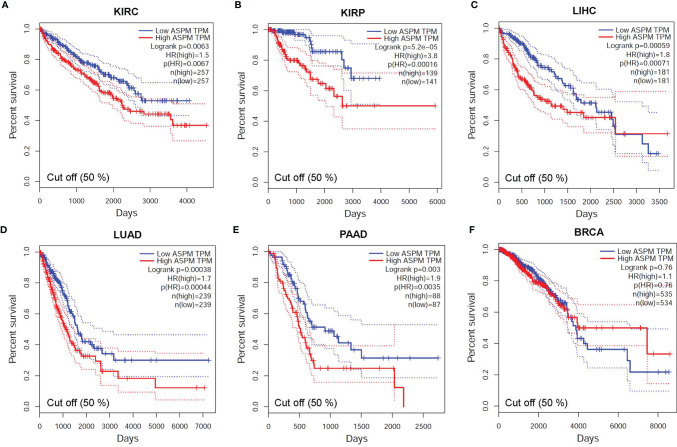
The relationship between the transcription level of ASPM and cancer prognosis. Survival curves showing association of ASPM expression with prognosis: overall survival (OS). For the accurate interpretation of ASPM expression detection results, we set the cut-off value as 50%. **(A)** KIRC (HR = 1.9, p = 0.00044), **(B)** KIRP (HR = 3.8, p = 0.00016) **(C)** LIHC (HR = 1.8, p = 0.00071), **(D)** LUAD (HR = 1.7, p = 0.00044), **(E)** PAAD (HR = 1.9, p = 0.00035), and **(F)** BRCA (HR = 1.1, p = 0.76). HR: Hazard Ratio.

To verify the search results of the OncoLnc database, we searched the GEPIA database to predict the relationship between the transcription level of *ASPM* and cancer prognosis (**Figure 5**). Hazard Ratio (HR) mainly used for survival analysis. The survival curves showed that high ASPM expression is associated with poor prognosis in KIRC (OS: HR = 2.6, p = 3.3e-16; RFS: HR = 3.2, p = 0), KIRP (OS: HR = 4.1, p = 1.4e-15; RFS: HR = 4.2, p = 0), LIHC (OS: HR = 1.8, p = 0.00072; RFS: HR = 1.6, p = 0.0031), LUAD (OS: HR = 1.7, p = 0.00044; RFS: HR = 2.4, p = 0), and PAAD (OS: HR = 1.2, p = 0.024; RFS: HR = 1.1, p = 0.18) (p < 0.05). Taken together, these results suggest that *ASPM* expression may serve as a potential prognostic indicator in KIRC, KIRP, LIHC, LUAD, and PAAD. However, there was no significant correlation between *ASPM* expression and the survival rates in BRCA patients.

### Relationship Between *ASPM* Expression and Tumor-Infiltrating Immune Cells

**Figure 6:**
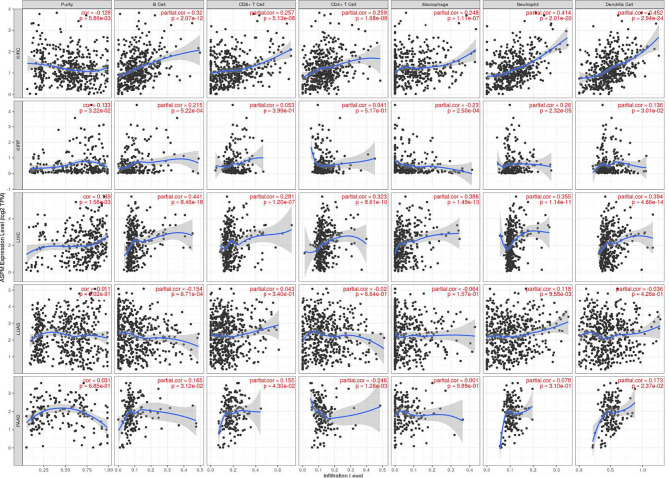
Relationship between ASPM expression and tumor-infiltrating immune cells using TIMER database. ASPM expression is related to immune cell infiltration (B cells, CD8^+^ T cells, CD4^+^ T cells macrophages, neutrophils, and DCs) in KIRC, KIRP, LIHC, LUAD, PAAD, and BRCA using the TIMER database. cor: Correlation.

Previous studies have shown that the number and activity of tumor infiltrating lymphocytes determine the survival time of several tumor patients (34, 35). Therefore, we aimed to determine whether *ASPM* expression is related to immune cell infiltration in KIRC, KIRP, LIHC, LUAD, PAAD, and BRCA using the TIMER database. Tumor purity is an important factor affecting immune infiltration analysis of clinical tumor samples (36). **Figure 6** shows that *ASPM* expression was significantly negatively correlated with tumor purity in KIRC (cor = -0.128, p = 5.86e-03), but positively correlated with tumor purity in KIRP (cor = -0.133, p = 3.22e-02), LIHC (cor = 0.169, p = 1.56e-03), and BRCA (cor = 0.177, p = 2.05e-08). There was no significant correlation between *ASPM* expression and tumor purity in LUAD and PAAD (p > 0.05). Interestingly, high *ASPM* expression in KIRC tissues markedly increased the infiltration of immune cells, such as B cells (cor = 0.32, p = 2.07e-12), CD8^+^ T cells (cor = 0.257, p = 5.13e-08), CD4^+^ T cells (cor = 0.259, p = 1.68e-08), macrophages (cor = 0.248, p = 1.11e-07), neutrophils (cor = 0.259, p = 1.68e-08), and DCs (cor = 0.452, p = 2.94e-24). In addition, we found that the expression of *ASPM* had a positive correlation with B cells (cor = 0.215, p = 5.22e-04), neutrophils (cor = 0.26, p = 2.32e-05), and DCs (cor = 0.136, p = 3.01e-02), but a negative correlation with macrophages (cor = -0.23, p = 2.5e-04). There was no significant correlation between *ASPM* expression and CD8^+^ T cells or CD4^+^ T cells (p > 0.05) in KIRP. For LIHC, the expression of ASPM was positively correlated with B cells (cor = 0.441, p = 8.46e-18), CD8^+^ T cells (cor = 0.281, p = 1.20e-17), CD4^+^ T cells (cor = 0.323, p = 8.61e-10), macrophages (cor = 0.386, p = 1.49e-13), neutrophils (cor = 0.355, p = 1.14e-11), and DCs (cor = 0.394, p = 4.66e-14). These findings implicate that *ASPM* plays a specific role in immune cell infiltration in KIRC, KIRP, and LIHC.

### Correlation Analysis Between the mRNA Levels of *ASPM* and Immune Cell Markers

To further verify the previous results, we investigated the correlation between the expression of *ASPM* and the state of immune cells based on the expression levels of immune marker genes using the TIMER database and immunohistochemistry in KIRC, KIRP, and LIHC tissues, including B cells, T cells, CD8^+^ T cells, monocytes, tumor-associated macrophages (TAMs), M1 and M2 macrophages, neutrophils, NK cells, and DCs (**Table 2**). Specifically, *ASPM* expression showed a significant correlation with the expression of markers related to specific immune cells, such as B cell, CD8^+^ T cells, and M2 macrophages in KIRC and LIHC. These results suggested that the expression of *ASPM* was related to B cell, CD8^+^ T cells, and M2 macrophages in KIRC and LIHC.

**Table 2:** The correlation between ASPM expression level and immune cell infiltration in KIRC and LIHC using the TIMER database.

		KIRC (N = 533)				LIHC (N = 371)			
		None		Purity		None		Purity	
		Cor	P-value	Cor	P-value	Cor	P-value	Cor	P-value
**B cell**	**CD19**	0.227	1.23e-07	0.198	***	0.233	5.68e-06	0.315	***
	**CD79A**	0.162	1.66e-04	0.140	2.51e-03	0.134	9.69e-03	0.265	***
**T cell (general)**	**CD3D**	0.339	8.04e-16	0.318	***	0.21	4.94e-05	0.336	***
	**CD3E**	0.348	1.37e-16	0.323	***	0.173	8.07e-04	0.339	***
	**CD2**	0.398	1.23e-21	0.379	***	0.175	7.48e-04	0.328	***
**CD8+ T cell**	**CD8A**	0.366	2.37e-18	0.355	***	0.182	4.37 e-04	0.303	***
	**CD8B**	0.310	2.53e-13	0.296	***	0.131	1.16e-02	0.241	***
**Monocyte**	**CD86**	0.396	2.03e-21	0.403	***	0.241	2.71e-06	0.399	***
	**CD115 (CSF1R)**	0.335	1.93e-15	0.318	***	0.106	4.1e-02	0.251	***
**TAM**	**CCL2**	-0.008	8.46e-01	-0.060	2.01e-01	0.036	4.89e-01	0.156	3.78e-03
	**CD68**	0.368	1.71e-18	0.379	***	0.167	1.25e-03	0.259	***
	**IL10**	0.341	5.65e-16	0.338	***	0.189	2.59e-04	0.305	***
**M1 Macrophage**	**INOS (NOS2)**	0.038	3.87e-01	0.012	7.96e-01	0.09	8.35e-02	0.098	6.98e-02
	**IRF5**	0.317	6.32e-14	0.315	***	0.424	1.32e-17	0.432	***
	**COX2(PTGS2)**	0.135	1.76e-03	0.115	1.36e-02	0.106	4.04e-02	0.249	***
**M2 Macrophage**	**CD163**	0.336	1.52e-15	0.339	***	0.093	7.37e-02	0.213	***
	**VSIG4**	0.320	4.06e-14	0.301	***	0.071	1.75e-01	0.189	***
	**MS4A4A**	0.325	1.31e-14	0.326	***	0.084	1.05e-01	0.219	***
**Neutrophils**	**CD66b (CEACAM8)**	0.021	6.32e-01	0.054	2.43e-01	0.095	6.77e-02	0.128	1.77e-02
	**CD11b (ITGAM)**	0.348	1.35e-16	0.329	***	0.232	6.97e-06	0.338	***
	**CCR7**	0.259	1.36e-09	0.239	***	0.094	7.07e-02	0.252	***
**Natural killer cell**	**KIR2DL1**	0.001	9.82e-01	-0.007	8.81e-01	-0.025	6.35e-01	-0.044	4.11e-01
	**KIR2DL3**	0.006	8.89e-01	0.005	9.09e-01	0.17	1.01e-03	0.2	1.85e-04
	**KIR3DL1**	-0.014	7.39e-01	0.001	9.83e-01	0.018	7.28e-01	0.047	3.82e-01
	**KIR3DL2**	0.029	5.04e-01	0.021	6.49e-01	0.114	2.85e-02	0.157	3.48e-03
	**KIR3DL3**	0.04	3.58e-01	0.025	5.99e-01	0.054	2.99e-01	0.063	2.43e-01
	**KIR2DS4**	-0.03	4.93e-01	-0.027	5.66e-01	0.054	2.96e-01	0.034	5.26e-01
**Dendritic cell**	**HLA-DPB1**	0.263	7.03e-10	0.262	***	0.129	1.29e-02	0.253	***
	**HLA-DQB1**	0.154	3.49e-04	0.137	3.10e-03	0.114	2.85e-02	0.233	***
	**HLA-DRA**	0.31	2.39e-13	0.324	***	0.163	1.69e-03	0.297	***
	**HLA-DPA1**	0.327	8.79e-15	0.34	***	0.141	6.72e-03	0.275	***
	**BDCA-1(CD1C)**	0.106	1.47e-02	0.086	6.46e-02	0.16	2.02e-03	0.279	***
	**CD11c (ITGAX)**	0.312	1.79e-13	0.31	***	0.292	1.16e-08	0.426	***
**Th1**	**T-bet (TBX21)**	0.198	3.9e-06	0.175	***	0.102	4.94e-02	0.212	***
	**STAT4**	0.387	1.74e-20	0.373	***	0.216	2.86e-05	0.292	***
	**STAT1**	0.515	1.7e-37	0.534	***	0.425	0e-00	0.487	***
	**IFN-γ (IFNG)**	0.428	3.51e-25	0.424	***	0.24	3.02e-06	0.331	***
	**TNF-α (TNF)**	0.257	1.75e-09	0.252	***	0.242	2.43e-06	0.376	***
**Th2**	**GATA3**	0.141	1.06e-03	0.116	1.28e-02	0.166	1.3e-03	0.303	***
	**STAT6**	0.1	2.08e-02	0.126	6.56e-03	0.187	2.88e-04	0.183	***
	**STAT5A**	0.302	9.89e-13	0.269	***	0.224	1.31e-05	0.293	***
	**IL13**	0.063	1.47e-01	0.056	2.27e-01	0.1	5.38e-02	0.114	3.35e-02
**Tfh**	**BCL6**	0.173	6.1e-05	0.173	***	0.199	1.14e-04	0.205	***
	**IL21**	0.227	1.25e-07	0.219	***	0.164	1.52e-03	0.194	***
**Th17**	**IL17A**	0.017	6.87e-01	-0.012	7.99e-01	0.137	8.45e-03	0.149	5.62e-03
**Treg**	**FOXP3**	0.433	1.01e-25	0.42	***	0.222	1.58e-05	0.298	***
	**CCR8**	0.472	6.94e-31	0.477	***	0.414	8.18e-17	0.521	***
	**STAT5B**	0.05	2.47e-01	0.058	2.13e-01	0.355	2.54e-12	0.348	***
	**TGFβ (TGFB1)**	0.271	1.92e-10	0.215	***	0.226	1.17e-05	0.329	***
**T cell exhaustion**	**PD-1 (PDCD1)**	0.348	1.24e-16	0.339	***	0.273	9.12e-08	0.382	***
	**CTLA4**	0.379	1.08e-19	0.368	***	0.277	6.05e-08	0.399	***
	**TIM-3 (HAVCR2)**	0.162	1.74e-04	0.154	***	0.241	2.73e-06	0.403	***
	**GZMB**	0.172	6.88e-05	0.141	2.43e-03	0.082	1.16e-01	0.155	3.97e-03

(*P < 0.05, **P <0.01, ***P < 0.001).

**Figure 7:**
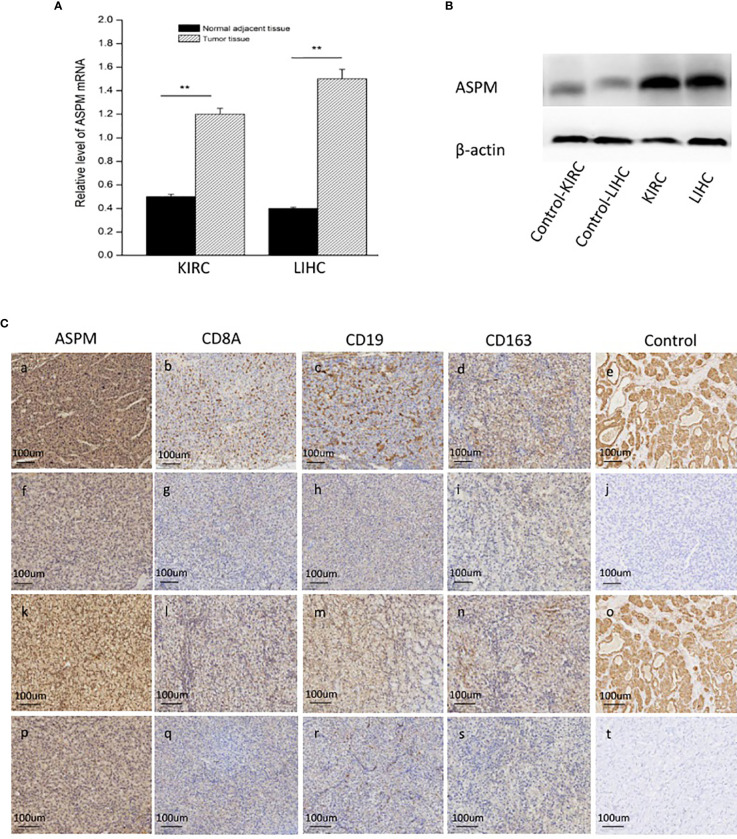
Expression analysis of ASPM in KIRC and LIHC tissues. **(A)** Relative level of ASPM mRNA using quantitative RT-PCR; **p < 0.01. **(B)** The expression of ASPM was analyzed by Western-blot analysis using a compound samples. **(C)** Tumor infiltration of B cells, CD8^+^ T cells, and M2 macrophages in KIRC and LIHC using immunohistochemistry. 20 diagnosed cases of KIRC and 20 diagnosed cases of LIHC samples for immunohistochemistry. We validate the relationship between ASPM expression and B cells (marker: CD19), CD8^+^ T cells (marker: CD8A), and M2 macrophages (marker: CD163), we performed immunohistochemistry to assess ASPM, CD19, CD8A, and CD163. Muscle and lymph nodes as control samples. **(a-d, f-i)** Tumor infiltration of B cells, CD8^+^ T cells, and M2 macrophages in KIRC. **(k-n, p-s)** Tumor infiltration of B cells, CD8^+^ T cells, and M2 macrophages in LIHC. **(a-d)** High expression of ASPM (+++), CD8A (++), CD19 (++), and CD163 (++) in KIRC. **(f-i)** Low expression of ASPM (+), CD8A (+), CD19 (+), and CD163 (+) in KIRC. **(k-n)** High expression of ASPM (+++), CD8A (++), CD19 (++), and CD163 (++) in LIHC. **(p-s)** Low expression of ASPM (+), CD8A (+), CD19 (+), and CD163 (+) in LIHC. **(e, o)** lymph nodes was used as a positive control in KIRC (+++) and LIHC (+++). **(j, t)** muscle was used as a negative control in KIRC (-) and LIHC (-). The expression density of ASPM, CD8A, CD19, and CD163 in KIRC and LIHC tissues were quantitated by scoring staining intensity, including negative (–) and weak (+) staining, moderate (++) and strong (+ + +) staining, respectively.

**Figure 8:**
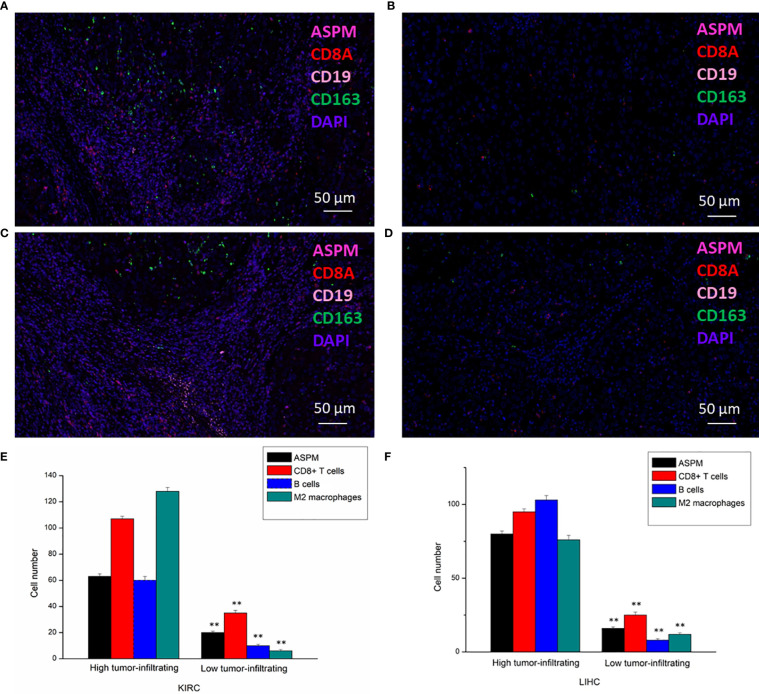
Detection of tumor-infiltrating levels of CD8^+^ T cells, B cells, and M2 macrophages in KIRC and LIHC using multiplex quantitative immunofluorescence. 20 diagnosed cases of KIRC and 20 diagnosed cases of LIHC samples for ultiplex quantitative immunofluorescence. Representative fluorescence images showing the detection of immune cells samples by simultaneous staining of DAPI, ASPM (rose red channel), CD8A (red channel), CD19 (pink channel), and CD163 (green channel) in KIRC **(A, B)** and LIHC **(C, D)**. CD8^+^ T cells marker: CD8A; B cells marker: CD19; M2 macrophages marker: CD163. **(E, F)**: Immune cell infiltration level at high or low expression of ASPM in KIRC and LIHC. **p < 0.01.

The transcription levels of *ASPM* were analyzed. The results of RT-qPCR showed that the expression of *ASPM* mRNA was significantly upregulated in KIRC and LIHC tissues (**Figure 7A**). Western blot results showed ASPM was highly expressed in KIRC and LIHC tissues (**Figure 7B**). Next, we further analyzed the correlation between ASPM expression and these markers by immunohistochemistry. The levels of the expression were quantitated by scoring staining intensity, including negative (–) and weak (+) staining, moderate (++) and strong (+++) staining, respectively. The infiltration of immune cells in lymph nodes was used as a positive control, while muscle was used as a negative control. Interestingly, the expression levels of CD19, CD8A, and CD163 in ASPM strong expression samples were higher than those in ASPM weak expression samples (**Figure 7C**). The results further revealed that CD19, CD8A, and CD163 tend to express in a positive correlation way, suggesting that the high expression of ASPM relates to high infiltration levels of B cell, CD8^+^ T cells, and M2 macrophages in KIRC and LIHC. In order to verify the above experimental results, we measured the tumor-infiltrating levels of B cells, CD8^+^ T cells, and M2 macrophages at high or low expression of ASPM in KIRC and LIHC using quantitative immunofluorescence. We found that ASPM expression relates to high infiltration of B cell, CD8^+^ T cells, and M2 macrophages in KIRC and LIHC (**Figure 8**). These findings strongly suggest that ASPM expression correlates with the infiltration of immune cells in KIRC and LIHC.

### *ASPM* Serves as an Independent Prognostic Marker in KIRC and LIHC

**Figure 9:**
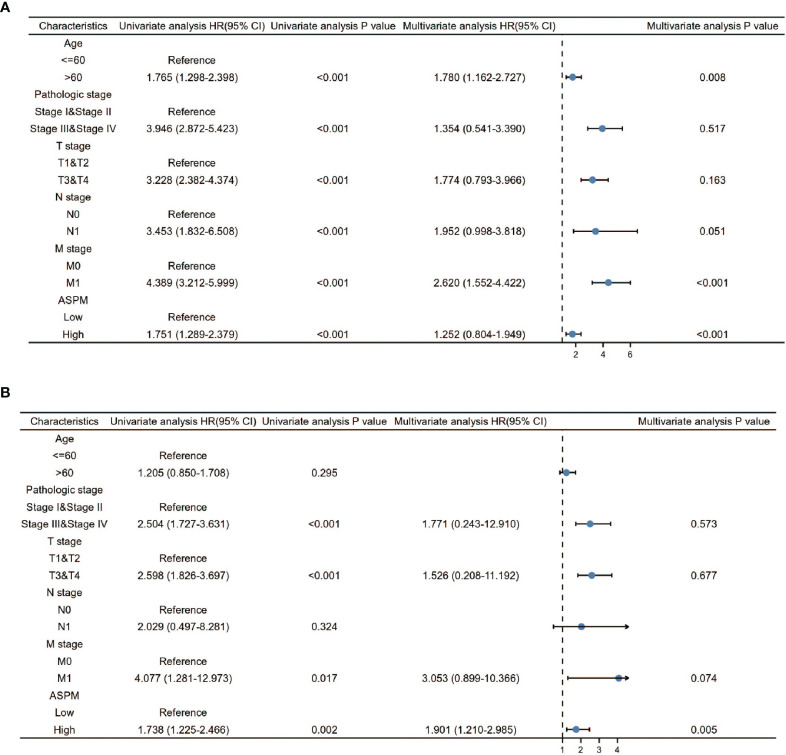
Univariate and Multivariate Cox analysis of ASPM and pathological parameters in KIRC **(A)** and LIHC **(B)**. 611 KIRC patients and 424 LIHC patients were used for analysis. Patients were divided into different subgroups according to, age, clinical stage, clinical TNM stage, and ASPM expression. For each subgroup, the prognostic performance of ASPM on overall survival was evaluated by Cox regression, and the results are presented as hazard ratio (HR). The bar represents the 95% confidence interval of HR.

Furthermore, for understanding the prognostic role of *ASPM* expression in KIRC and LIHC, the Cox proportional hazard regression model was employed to analyze prognostic factors. A Cox univariate survival analysis indicated that age (p < 0.001), pathologic stage (p < 0.001), TNM stages (p < 0.001) were significant parameters that affect the survival time of KIRC patients (**Figure 9A**). In addition, we found that *ASPM* expression was significant related with pathologic stage (p < 0.001) and T stage (p < 0.001) in LIHC (**Figure 9B**). For verifying the prognostic value of *ASPM* in KIRC and LIHC, we performed the multivariate analysis. The results showed that *ASPM* expression was independently associated with the OS time in KIRC and LIHC patients.

## Discussion

Previous studies have shown that ASPM is highly expressed in a variety of cancers and is related to a poor clinical prognosis and recurrence (37). Accumulating evidence suggests that ASPM is a regulator of cell stemness, it enhances stem cell phenotype in prostate cancer cells by enhancing Wnt/β-catenin signaling, thereby promoting tumor aggressiveness (38). ASPM acts as an immune-related gene in bladder cancer (23). However, the potential correlation of ASPM expression with immune cell infiltration in other cancers remained unclear. Overexpression of the ASPM gene is associated with aggressiveness and poor outcome in bladder cancer (39). To date, the clinical relevance and prognostic significance of ASPM in other cancer remain unknown. We hypothesized that ASPM may be associated with immune infiltration in other cancers. The study demonstrated that the expression of ASPM correlated with the prognosis of several human cancers, including KIRC and LIHC. The survival rates of patients with various types were related to the expression level of ASPM. Furthermore, we predicted the association between the expression level of ASPM and the infiltration levels of different immune cells in cancer between various and types. Our study suggests that ASPM is a potential prognostic biomarker for KIRC and LIHC.

The mRNA levels of *ASPM* in various types of cancer tissues and their corresponding normal tissues were analyzed using the TIMER and DriverDB databases. The results revealed that *ASPM* expression was significantly upregulated in most cancer tissues. To gain deeper insight into the biological interaction network of ASPM, we analyzed the functional protein association network by using the STRING database and performed biological analyses of the identified genes *via* the Enrichr online database. We found that ASPM was co-expressed with KIF11, CDK1, BUB1, NCAPG, CENPE, TTK, KIF23, DLGAP5, CDC20, and CCNA2. Daigo K et al. revealed that KIF11 could be a potential prognostic biomarker and therapeutic target for oral cancer (17). KIF11 plays an important role in the cancer stem cells of esophageal squamous cell carcinoma and colorectal cancer (40). KIF11 was primarily involved in cell cycle, TME alteration and tumor-infiltrating immune cells proportions (41, 42). The cell cycle-related proteins CDK1 and BUB1 are significantly overexpressed in pancreatic ductal adenocarcinoma tissues and may be prognostic biomarkers (43). NCAPG is a novel biomarker of prognosis and is associated with immune cell infiltration in the tumor microenvironment, and it promotes cell proliferation and inhibits apoptosis by activating the PI3K/Akt/foxo4 pathway in hepatocellular carcinoma (44, 45). Xiong C et al. demonstrates that CDC20 may be an immune-associated therapeutic target in hepatocellular carcinoma because of its correlation with immune infiltration (46). These results together showed that ASPM may be associated with immune cell infiltration in cancer. All these findings indicated that targeting ASPM could be a promising strategy and warrants further investigation. Therefore, we speculate that ASPM may be related to the occurrence and development of cancer. Previous studies have reported that increased ASPM expression may predict poor biochemical recurrence-free survival in prostate cancer patients (14). Overexpression of the ASPM gene is associated with a poor prognosis in bladder cancer (17). Our data showed that ASPM was highly expressed in KIRC and LIHC tissues. To determine whether ASPM expression is associated with survival outcomes in patients with KIRC, KIRP, LIHC, LUAD, and PAAD, we analyzed the correlation between the expression of *ASPM* and prognosis using samples from the OncoLnc and GEPIA databases, high *ASPM* expression was associated with a poor prognosis. These results suggest that *ASPM* expression may serve as a prognostic indicator in KIRC, KIRP, LIHC, LUAD, and PAAD.

Several studies have shown that immune cell infiltration is a prognostic marker of cancer progression (47–49). Therefore, we hypothesized that ASPM expression was associated with immune infiltration in KIRC, KIRP, LIHC, LUAD, and PAAD, and could be a potential marker of the tumor immune microenvironment. The link between high ASPM expression and tumour infiltrating immune cell cells has already been shown in bladder cancer (23). In our study, we found that ASPM expression positively correlated with immune cell infiltration in KIRC and LIHC, particularly with B cells, CD8^+^ T cells, and M2 macrophages in KIRC and LIHC. To the best of our knowledge, this study is the first to evaluate the association between ASPM expression and the levels of immune infiltration in KIRC and LIHC. Our results demonstrated a positive correlation between ASPM expression and the levels of B cells (marker: CD19), CD8^+^ T cells (marker: CD8A), and M2 macrophages (marker: CD163) infiltration. The results are consistent with our hypothesis. Macrophages are a group of differentiated immune cells and classify as M1 macrophages and M2 macrophages, they play an important role in development, homeostasis, and immunity (50, 51). CD163 is a marker of M2 macrophages and plays the role of regulating tumor infiltration (52, 53). Our studies observed that an increase in ASPM expression was correlated with the M2 macrophage marker. It suggests that there is a specific correlation between ASPM expression and the immune infiltrating of M2 macrophages. Furthermore, ASPM expression was significantly correlated with B cells markers such as CD19. CD19-targeted chimeric antigen receptor-modified T cell immunotherapy has demonstrated impressive results in B-cell malignancies (54). Studies have reported a positive correlation between elevated CD8^+^ T cells in the tumor microenvironment and a good prognosis of cancer (55). In this study, we found that the expression of ASPM was related to CD8^+^ T cells. This suggests a role for ASPM in regulating the tumor-infiltration of B cells, CD8^+^ T cells and M2 macrophages. In addition, we further analyzed the effects of death risk factors on breast cancer by univariate/multivariate Cox regression analysis proved that high expression of *ASPM* can be used as an independent prognostic factor for KIRC and LIHC. As a potential prognostic marker, ASPM deserves further clinical verification. Hence, our study suggests that *ASPM* is a potential biomarker for KIRC and LIHC prognosis and the status of tumor immunity. The underlying mechanism between ASPM expression and tumor-infiltrating immune cells should be further explored.

Since this study was based on data obtained from multiple online databases, there were some limitations to this study. A large sample size is needed to reliably interpret the data. We will further conduct animal experiments to confirm the relationship between ASPM in KIRC and LIHC and immune cell infiltration into the tumor microenvironment.

In conclusion, our study indicates that *ASPM* has prognostic value in KIRC and LIHC, because its overexpression is associated with a poor prognosis and immune cell infiltration. ASPM may play an important role in the microenvironment of KIRC and LIHC by regulating tumor infiltration of immune cells. Therefore, our analysis provides an integrated understanding of the potential role of ASPM in KIRC and LIHC and its use as a prognostic target to modulate the antitumor immune response.

## Data Availability Statement

Publicly available datasets were analyzed in this study. This data can be found here: TIMER database (https://cistrome.shinyapps.io/timer/); UALCAN database (http://ualcan.path.uab.edu/); STRING (https://string-db.org/); Enrichr database (http://amp.pharm.mssm.edu/Enrichr); OncoLnc (http://www.oncolnc.org/); GEPIA (http://gepia.cancer-pku.cn/index.html).

## Ethics Statement

The studies involving human participants were reviewed and approved by The Ethics Committee of Shenzhen Second People’s Hospital in accordance with the principles of the Declaration of Helsinki. The patients/participants provided their written informed consent to participate in this study. Written informed consent was obtained from the individual(s) for the publication of any potentially identifiable images or data included in this article.

## Author Contributions

TD and YL conceived the project and wrote the manuscript. JZ performed part of the experiments. QH participated in data analysis and participated in discussion and language editing. YT reviewed the manuscript and supervised this project. All authors contributed to the article and approved the submitted version.

## Funding

This work was supported by National Natural Science Foundation of China (82002936); Guangdong Natural Science Foundation (2020A1515010347); Shenzhen Science and Technology Innovation Committee (JCYJ20190806163209126, JCYJ20180228162815750, JCYJ20190806164203496 and ZDSYS20190902093401689); the Major Scientific and Technological Project of Guangdong Province (2020A1515110419); Shenzhen Municipal Government of China (RCBS20200714114856269, JCYJ20210324102807019); China Postdoctoral Science Foundation Funded Project (2021T140478).

## Conflict of Interest

The authors declare that the research was conducted in the absence of any commercial or financial relationships that could be construed as a potential conflict of interest.

## Publisher’s Note

All claims expressed in this article are solely those of the authors and do not necessarily represent those of their affiliated organizations, or those of the publisher, the editors and the reviewers. Any product that may be evaluated in this article, or claim that may be made by its manufacturer, is not guaranteed or endorsed by the publisher.

## References

[1] Kurozumi S Fujii T Matsumoto H Inoue K Kurosumi M Horiguchi J. Significance of Evaluating Tumor-Infiltrating Lymphocytes (TILs) and Programmed Cell Death-Ligand 1 (PD-L1) Expression in Breast Cancer. Med Mol Morphol (2017) 50(4):185–94. doi: 10.1007/s00795-017-0170-y 28936553

[2] Lee N Zakka LR Mihm MC Jr Schatton T. Tumour-Infiltrating Lymphocytes in Melanoma Prognosis and Cancer Immunotherapy. Pathology (2016) 48(2):177–87. doi: 10.1016/j.pathol.2015.12.006 27020390

[3] Liu X Wu S Yang Y Zhao M Zhu G Hou Z. The Prognostic Landscape of Tumor-Infiltrating Immune Cell and Immunomodulators in Lung Cancer. BioMed Pharmacother (2017) 95:55–61. doi: 10.1016/j.biopha.2017.08.003 28826097

[4] Santoiemma PP Powell DJ Jr. Tumor Infiltrating Lymphocytes in Ovarian Cancer. Cancer Biol Ther (2015) 16(6):807–20. doi: 10.1080/15384047.2015.1040960 PMC4622931 25894333

[5] Wang K Shen T Siegal GP Wei S. The CD4/CD8 Ratio of Tumor-Infiltrating Lymphocytes at the Tumor-Host Interface has Prognostic Value in Triple-Negative Breast Cancer. Hum Pathol (2017) 69:110–7. doi: 10.1016/j.humpath.2017.09.012 28993275

[6] Kong JC Guerra GR Pham T Mitchell C Lynch AC Warrier SK. Prognostic Impact of Tumor-Infiltrating Lymphocytes in Primary and Metastatic Colorectal Cancer: A Systematic Review and Meta-Analysis. Dis Colon Rectum (2019) 62(4):498–508. doi: 10.1097/DCR.0000000000001332 30844974

[7] Zheng X Song X Shao Y Xu B Hu W Zhou Q. Prognostic Role of Tumor-Infiltrating Lymphocytes in Esophagus Cancer: A Meta-Analysis. Cell Physiol Biochem (2018) 45(2):720–32. doi: 10.1159/000487164 29414812

[8] Ding W Xu X Qian Y Xue W Wang Y Du J. Prognostic Value of Tumor-Infiltrating Lymphocytes in Hepatocellular Carcinoma: A Meta-Analysis. Med (Baltimore) (2018) 97(50):e13301. doi: 10.1097/MD.0000000000013301 PMC6320107 30557978

[9] Uryvaev A Passhak M Hershkovits D Sabo E Bar-Sela G. The Role of Tumor-Infiltrating Lymphocytes (TILs) as a Predictive Biomarker of Response to Anti-PD1 Therapy in Patients With Metastatic non-Small Cell Lung Cancer or Metastatic Melanoma. Med Oncol (2018) 35(3):25. doi: 10.1007/s12032-018-1080-0 29388007

[10] Kashiwagi S Asano Y Goto W Takada K Takahashi K Hatano T. Using TILs to Predict Therapeutic Effect of Chemotherapy (Pertuzumab, Trastuzumab, Docetaxel) on HER2-Positive Breast Cancer. Anticancer Res (2017) 37(10):5623–30. doi: 10.21873/anticanres.11997 28982879

[11] Fernandez-Poma SM Salas-Benito D Lozano T Casares N Riezu-Boj JI Mancheño U. Expansion of Tumor-Infiltrating CD8+ T Cells Expressing PD-1 Improves the Efficacy of Adoptive T-Cell Therapy. Cancer Res (2017) 77(13):3672–84. doi: 10.1158/0008-5472.CAN-17-0236 28522749

[12] Fish JL Kosodo Y Enard W Pääbo S Huttner WB. Aspm Specifically Maintains Symmetric Proliferative Divisions of Neuroepithelial Cells. Proc Natl Acad Sci U S A (2006) 103(27):10438–43. doi: 10.1073/pnas.0604066103 PMC1502476 16798874

[13] Woods CG Bond J Enard W. Autosomal Recessive Primary Microcephaly (MCPH): A Review of Clinical, Molecular, and Evolutionary Findings. Am J Hum Genet (2005) 76(5):717–28. doi: 10.1086/429930 PMC1199363 15806441

[14] Buchman JJ Durak O Tsai LH. ASPM Regulates Wnt Signaling Pathway Activity in the Developing Brain. Genes Dev (2011) 25:1909–14. doi: 10.1101/gad.16830211 PMC3185963 21937711

[15] Xie JJ Zhuo YJ Zheng Y Mo RJ Liu ZZ Li BW. High Expression of ASPM Correlates With Tumor Progression and Predicts Poor Outcome in Patients With Prostate Cancer. Int Urol Nephrol (2017) 49(5):817–23. doi: 10.1007/s11255-017-1545-7 28213802

[16] Tang J Lu M Cui Q Zhang D Kong D Liao X. Overexpression of ASPM, CDC20, and TTK Confer a Poorer Prognosis in Breast Cancer Identified by Gene Co-Expression Network Analysis. Front Oncol (2019) 9:310. doi: 10.3389/fonc.2019.00310 31106147 PMC6492458

[17] Xu Z Zhang Q Luh F Jin B Liu X. Overexpression of the ASPM Gene is Associated With Aggressiveness and Poor Outcome in Bladder Cancer. Oncol Lett (2019) 17(2):1865–76. doi: 10.3892/ol.2018.9762 PMC6341836 30675249

[18] Pai VC Hsu CC Chan TS Liao WY Chuu CP Chen WY. ASPM Promotes Prostate Cancer Stemness and Progression by Augmenting Wnt-Dvl-3-β-Catenin Signaling. Oncogene (2019) 38(8):1340–53. doi: 10.1038/s41388-018-0497-4 30266990

[19] Chen X Huang L Yang Y Chen S Sun J Ma C. ASPM Promotes Glioblastoma Growth by Regulating G1 Restriction Point Progression and Wnt-β-Catenin Signaling. Aging (2020) 12(1):224–41. doi: 10.18632/aging.102612 PMC6977704 31905171

[20] Khadirnaikar S Kumar P Pandi SN Malik R Dhanasekaran SM Shukla SK. Immune Associated LncRNAs Identify Novel Prognostic Subtypes of Renal Clear Cell Carcinoma. Mol Carcinog. (2019) 58(4):544–53. doi: 10.1002/mc.22949 30520148

[21] He R Wang L Li J Ma L Wang F Wang Y. Integrated Analysis of a Competing Endogenous RNA Network Reveals a Prognostic Signature in Kidney Renal Papillary Cell Carcinoma. Front Cell Dev Biol (2020) 8:612924. doi: 10.3389/fcell.2020.612924 33344459 PMC7744790

[22] Wu L Quan W Luo Q Pan Y Peng D Zhang G. Identification of an Immune-Related Prognostic Predictor in Hepatocellular Carcinoma. Front Mol Biosci (2020) 7:567950. doi: 10.3389/fmolb.2020.567950 33195412 PMC7542239

[23] Xu Y Wu G Li J Li J Ruan N Ma L. Screening and Identification of Key Biomarkers for Bladder Cancer: A Study Based on TCGA and GEO Data. BioMed Res Int (2020) 2020:8283401. doi: 10.1155/2020/8283401 32047816 PMC7003274

[24] Li T Fan J Wang B Traugh N Chen Q Liu JS. TIMER: A Web Server for Comprehensive Analysis of Tumor-Infiltrating Immune Cells. Cancer Res (2017) 77(21):e108–10. doi: 10.1158/0008-5472.CAN-17-0307 PMC6042652 29092952

[25] Li B Severson E Pignon JC Zhao H Li T Novak J. Comprehensive Analyses of Tumor Immunity: Implications for Cancer Immunotherapy. Genome Biol (2016) 17(1):174. doi: 10.1186/s13059-016-1028-7 27549193 PMC4993001

[26] Chandrashekar DS Bashel B Balasubramanya SAH Creighton CJ Ponce Rodriguez I Chakravarthi BVSK. UALCAN: A Portal for Facilitating Tumor Subgroup Gene Expression and Survival Analyses. Neoplasia (2017) 19:649–58. doi: 10.1016/j.neo.2017.05.002 PMC5516091 28732212

[27] Crosara KTB Moffa EB Xiao Y Siqueira WL. Merging in-Silico and *In Vitro* Salivary Protein Complex Partners Using the STRING Database: A Tutorial. J PROTEOMICS (2018) 171:87–94. doi: 10.1016/j.jprot.2017.08.002 28782718

[28] Gao L Li SH Tian YX Zhu QQ Chen G Pang Y. Role of Downregulated miR-133a-3p Expression in Bladder Cancer: A Bioinformatics Study. Onco Targets Ther (2017) 10:3667–83. doi: 10.2147/OTT.S137433 PMC5530854 28790856

[29] Kuleshov MV Jones MR Rouillard AD Fernandez NF Duan Q Wang Z. Enrichr: A Comprehensive Gene Set Enrichment Analysis Web Server 2016 Update. Nucleic Acids Res (2016) 44(W1):W90–7. doi: 10.1093/nar/gkw377 PMC4987924 27141961

[30] Cai C Wang W Tu Z. Aberrantly DNA Methylated-Differentially Expressed Genes and Pathways in Hepatocellular Carcinoma. J Cancer (2019) 10(2):355–66. doi: 10.7150/jca.27832 PMC6360310 30719129

[31] Lu Y Li C Chen H Zhong W. Identification of Hub Genes and Analysis of Prognostic Values in Pancreatic Ductal Adenocarcinoma by Integrated Bioinformatics Methods. Mol Biol Rep (2018) 45(6):1799–807. doi: 10.1007/s11033-018-4325-2 30173393

[32] Wang W Ren S Wang Z Zhang C Huang J. Increased Expression of TTC21A in Lung Adenocarcinoma Infers Favorable Prognosis and High Immune Infiltrating Level. Int Immunopharmacol (2020) 78:106077. doi: 10.1016/j.intimp.2019.106077 31812070

[33] Gil M Kim KE. Interleukin-18 Is a Prognostic Biomarker Correlated With CD8+ T Cell and Natural Killer Cell Infiltration in Skin Cutaneous Melanoma. J Clin Med (2019) 8(11):1993. doi: 10.3390/jcm8111993 PMC6912818 31731729

[34] Japanese Gastric Cancer Association. Japanese Gastric Cancer Treatment Guidelines 2014 (Ver. 4). Gastric Cancer (2017) 20:1–19. doi: 10.1007/s10120-016-0622-4 PMC5215069 27342689

[35] Ohtani H. Focus on TILs: Prognostic Significance of Tumor Infiltrating Lymphocytes in Human Colorectal Cancer. Cancer Immun (2007) 7:4. 17311363 PMC2935759

[36] Yoshihara K Shahmoradgoli M Martínez E Vegesna R Kim H TorresGarcia W. Inferring Tumour Purity and Stromal and Immune Cell Admixture From Expression Data. Nat Commun (2013) 4:2612. doi: 10.1038/ncomms3612 24113773 PMC3826632

[37] Zhou JW Wang H Sun W Han NN Chen L. ASPM is a Predictor of Overall Survival and has Therapeutic Potential in Endometrial Cancer. Am J Transl Res (2020) 12(5):1942–53. PMC7270042 32509189

[38] Timaner M Shaked Y. Elucidating the Roles of ASPM Isoforms Reveals a Novel Prognostic Marker for Pancreatic Cancer. J Pathol (2020) 250(2):123–5. doi: 10.1002/path.5355 31595972

[39] Kouprina N Pavlicek A Collins NK Nakano M Noskov VN Ohzeki J. The Microcephaly ASPM Gene is Expressed in Proliferating Tissues and Encodes for a Mitotic Spindle Protein. Hum Mol Genet (2005) 14(15):2155–65. doi: 10.1093/hmg/ddi220 15972725

[40] Daigo K Takano A Thang PM Yoshitake Y Shinohara M Tohnai I. Characterization of KIF11 as a Novel Prognostic Biomarker and Therapeutic Target for Oral Cancer. Int J Oncol (2018) 52(1):155–65. doi: 10.3892/ijo.2017.4181 PMC5743338 29115586

[41] Imai T Oue N Sentani K Sakamoto N Uraoka N Egi H. KIF11 Is Required for Spheroid Formation by Oesophageal and Colorectal Cancer Cells. Anticancer Res (2017) 37(1):47–55. doi: 10.21873/anticanres.11287 28011472

[42] Li Z Yu B Qi F Li F. KIF11 Serves as an Independent Prognostic Factor and Therapeutic Target for Patients With Lung Adenocarcinoma. Front Oncol (2021) 11:670218. doi: 10.3389/fonc.2021.670218 33968780 PMC8103954

[43] Piao J Zhu L Sun J Li N Dong B Yang Y. High Expression of CDK1 and BUB1 Predicts Poor Prognosis of Pancreatic Ductal Adenocarcinoma. Gene (2019) 701:15–22. doi: 10.1016/j.gene.2019.02.081 30898709

[44] Zhou Y Fan Y Mao Y Lou M Liu X Yuan K. NCAPG is a Prognostic Biomarker of Immune Infiltration in non-Small-Cell Lung Cancer. biomark Med (2022) 6(7). doi: 10.2217/bmm-2021-1090 35199566

[45] Gong C Ai J Fan Y Gao J Liu W Feng Q. NCAPG Promotes The Proliferation Of Hepatocellular Carcinoma Through PI3K/AKT Signaling. Onco Targets Ther (2019) 12:8537–52. doi: 10.2147/OTT.S217916 PMC6801502 31802891

[46] Xiong C Wang Z Wang G Zhang C Jin S Jiang G. Identification of CDC20 as an Immune Infiltration-Correlated Prognostic Biomarker in Hepatocellular Carcinoma. Invest New Drugs (2021) 39(5):1439–53. doi: 10.1007/s10637-021-01126-1 33942202

[47] Oda K Kato K Nakamura M Jotatsu T Noguchi S Kawanami T. Surface Marker Profiles on Lung Lymphocytes may Predict the Mechanism of Immune-Mediated Pneumonitis Triggered by Tumor-Infiltrating Lymphocytes in Lung Cancer Patients Treated With Pembrolizumab. Lung Cancer (2018) 118:171–2. doi: 10.1016/j.lungcan.2018.02.012 29496324

[48] Shimizu S Hiratsuka H Koike K Tsuchihashi K Sonoda T Ogi K. Tumor-Infiltrating CD8+ T-Cell Density is an Independent Prognostic Marker for Oral Squamous Cell Carcinoma. Cancer Med (2019) 8(1):80–93. doi: 10.1002/cam4.1889 30600646 PMC6346233

[49] Horvath S Zhang B Carlson M Lu KV Zhu S Felciano RM. Analysis of Oncogenic Signaling Networks in Glioblastoma Identifies ASPM as a Molecular Target. Proc Natl Acad Sci U S A (2006) 103(46):17402–7. doi: 10.1073/pnas.0608396103 PMC1635024 17090670

[50] Jayasingam SD Citartan M Thang TH Mat Zin AA Ang KC Ch’ng ES. Evaluating the Polarization of Tumor-Associated Macrophages Into M1 and M2 Phenotypes in Human Cancer Tissue: Technicalities and Challenges in Routine Clinical Practice. Front Oncol (2019) 9:1512. doi: 10.3389/fonc.2019.01512 32039007 PMC6992653

[51] Huo Q Li Z Cheng L Yang F Xie N. SIRT7 Is a Prognostic Biomarker Associated With Immune Infiltration in Luminal Breast Cancer. Front Oncol (2020) 10:621. doi: 10.3389/fonc.2020.00621 32528869 PMC7247806

[52] Rao J Wu X Zhou X Deng R Ma Y. TMEM205 Is an Independent Prognostic Factor and Is Associated With Immune Cell Infiltrates in Hepatocellular Carcinoma. Front Genet (2020) 11:575776. doi: 10.3389/fgene.2020.575776 33193690 PMC7592400

[53] Pan Y Lu F Fei Q Yu X Xiong P Yu X. Single-Cell RNA Sequencing Reveals Compartmental Remodeling of Tumor-Infiltrating Immune Cells Induced by Anti-CD47 Targeting in Pancreatic Cancer. J Hematol Oncol (2019) 12(1):124. doi: 10.1186/s13045-019-0822-6 31771616 PMC6880569

[54] Hirayama AV Turtle CJ. Toxicities of CD19 CAR-T Cell Immunotherapy. Am J Hematol (2019) 94(S1):S42–9. doi: 10.1002/ajh.25445 30784102

[55] Maimela NR Liu S Zhang Y. Fates of CD8+ T Cells in Tumor Microenvironment. Comput Struct Biotechnol J (2018) 17:1–13. doi: 10.1016/j.csbj.2018.11.004 30581539 PMC6297055

