# Corrigendum: ASPM is a prognostic biomarker and correlates with immune infiltration in kidney renal clear cell carcinoma and liver hepatocellular carcinoma

**DOI:** 10.3389/fonc.2022.979968

**Published:** 2022-08-22

**Authors:** Tingting Deng, Yang Liu, Jialang Zhuang, Yizhe Tang, Qin Huo

**Affiliations:** Department of Otolaryngology and Geriatric Medicine, Guangdong Key Laboratory of Systems Biology and Synthetic Biology for Urogenital Tumors, Shenzhen Second People's Hospital, First Affiliated Hospital of Shenzhen University, Health Science Center, Shenzhen University, Shenzhen, China

**Keywords:** ASPM, gene expression, prognosis, tumor-infiltrating, biomarker

In the published article, there was an error in the PDF page header. Instead of “Liu et al”, it should be “Deng et al.”.

In the published article, there was an error in affiliation 1. Instead of “^1^Department of Otolaryngology and Geriatric Medicine, Biobank, Shenzhen Institute of Translational Medicine, The First Affiliated Hospital of Shenzhen University Health Science Center, Shenzhen Second People’s Hospital, Shenzhen, China, ^2^Guangdong Key Laboratory of Systems Biology and Synthetic Biology for Urogenital Tumors, Shenzhen Second People’s Hospital, Shenzhen, China”, it should be “Department of Otolaryngology and Geriatric Medicine, Guangdong Key Laboratory of Systems Biology and Synthetic Biology for Urogenital Tumors, Shenzhen Second People’s Hospital, First Affiliated Hospital of Shenzhen University, Health Science Center, Shenzhen University, Shenzhen, China”.

In the published article, there was an error in the Funding statement. One funding source was carelessly missed with the code “ZDSYS20190902093401689”. The funding statement for the Shenzhen STIC Research Foundation should be combined with Shenzhen Science and Technology Innovation Committee since they stand for the same funding agency. The correct Funding statement appears below.

## Funding

This work was supported by National Natural Science Foundation of China (82002936); Guangdong Natural Science Foundation (2020A1515010347); Shenzhen Science and Technology Innovation Committee (JCYJ20190806163209126, JCYJ20180228162815750, JCYJ20190806164203496 and ZDSYS20190902093401689); the Major Scientific and Technological Project of Guangdong Province (2020A1515110419); Shenzhen Municipal Government of China (RCBS20200714114856269, JCYJ20210324102807019); China Postdoctoral Science Foundation Funded Project (2021T140478).

The authors apologize for this error and state that this does not change the scientific conclusions of the article in any way. The original article has been updated.

In the published article, there was an error in the Methods section, Patient Samples & Immunohistochemistry subsections. The second phrase of “20 diagnosed cases of KIRC and 20 control samples” in the same paragraph was a repeat typo and should be corrected as “20 diagnosed cases of LIHC and 20 control samples”.

Two same corrections have been made to Methods, Patient Samples *&* Immunohistochemistry subsections. This sentence previously stated “as well as 20 diagnosed cases of KIRC and 20 control samples (muscle, lymph nodes)”. In should instead be written as follows: “as well as 20 diagnosed cases of LIHC and 20 control samples (muscle, lymph nodes)”. The patient sample information is correctly displayed in the main text and figures in the published article.

The authors apologize for these errors and state that this does not change the scientific conclusions of the article in any way. The original article has been updated.

## Publisher’s note

All claims expressed in this article are solely those of the authors and do not necessarily represent those of their affiliated organizations, or those of the publisher, the editors and the reviewers. Any product that may be evaluated in this article, or claim that may be made by its manufacturer, is not guaranteed or endorsed by the publisher.

